# PARP inhibition and the radiosensitizing effects of the PARP inhibitor ABT-888 in *in vitro* hepatocellular carcinoma models

**DOI:** 10.1186/1471-2407-14-603

**Published:** 2014-08-20

**Authors:** Clément Guillot, Vincent Favaudon, Zdenko Herceg, Charlotte Sagne, Sylvie Sauvaigo, Philippe Merle, Janet Hall, Isabelle Chemin

**Affiliations:** UMR INSERM U1052 CNRS 5286, CRCL, 151 Cours A Thomas, Lyon, F-69008 France; Université Lyon-1, Villeurbanne, F-69622 France; Institut Curie, Bats 110–112 Centre Universitaire, Orsay, F-91405 France; Inserm U612, Bats 110–112 Centre Universitaire, Orsay, F-91405 France; International Agency for Research on Cancer, 150 cours Albert Thomas, F-69424 Lyon Cedex 03, France; Laboratoire Lésions des Acides Nucléiques, CEA, DSM/INAC/SCIB, UMR-E3 CEA/UJF-Grenoble 1, 17 rue des Martyrs, Grenoble, F-38054 France; Hospices Civils de Lyon, Service d’Hépatologie et de Gastroentérologie, Groupement Hospitalier Lyon Nord, Lyon, France

**Keywords:** Hepatocellular carcinoma, Poly(ADP-ribose) polymerases inhibitors, Radiation therapy

## Abstract

**Background:**

Hepatocellular carcinoma is the third cause of cancer related death for which new treatment strategies are needed. Targeting DNA repair pathways to sensitize tumor cells to chemo- or radiotherapy is under investigation for the treatment of several cancers with poly(ADP-ribose) polymerase (PARP) inhibitors showing great potential. The aim of this preclinical study was to evaluate the expression of *PARP* and *PARG* genes in a panel of liver cancer cell lines and primary human hepatocytes, their DNA repair capacity and assess the impact on cell survival of PARP inhibitors alone and in combination with radiotherapy.

**Methods:**

Quantitative PCR was used to measure *PARP-1*, -*2*, -*3* and *PARG* mRNA levels and western blotting for PARP-1 protein expression and ADP-ribose polymer formation after exposure of cells to doxorubicin, a topoisomerase II poison. DNA repair capacity was assessed using an *in vitro* DNA lesion excision/synthesis assay and the effects on cell killing of the PARP inhibitor ABT-888 alone and in combination with ionizing radiation using clonogenic survival.

**Results:**

Although a wide range in expression of the PARPs and PARG was found correlations between *PARP-1* and *PARP-2* mRNA levels and *PARP-1* mRNA and protein levels were noted. However these expression profiles were not predictive of PARP activity in the different cell lines that also showed variability in excision/synthesis repair capacity. 4 of the 7 lines were sensitive to ABT-888 alone and the two lines tested showed enhanced radiosensitivity in the presence of ABT-888.

**Conclusions:**

PARP inhibitors combined with radiotherapy show potential as a therapeutic option for hepatocellular carcinoma.

## Background

Hepatocellular carcinoma (HCC) is the third cause of cancer related death worldwide with an overall ratio of mortality to incidence of 0.93 [1]. Due to the often late diagnosis, possible curative therapies such as hepatic resection, liver transplantation and percutaneous ablation can be offered to only a limited number of patients [2] highlighting the need for the development of new treatment strategies. Modern technologies have allowed the emergence of new techniques in the field of radiation therapy. For example, three-dimensional conformal radiotherapy has the potential to accurately deliver high doses of radiation within a well-defined HCC tumor volume while sparing the surrounding non-tumor liver parenchyma and the adjacent organs, therefore limiting radiation-induced complications [3]. This method has shown promising results with an 80% complete response for small-size HCC nodules in patients non-eligible for standard curative therapies [4].

Molecules targeting DNA repair pathways have shown great potential to sensitize tumor cells to both chemo- and radiotherapy and increase their cytotoxicity [5]. Inhibitors of poly(ADP-ribose) polymerases (PARPs) fall into this category. Poly(ADP-ribosyl)ation is carried out by several members of the PARP family, of which PARP-1 is the most prevalent. This ubiquitous post-translational modification in mammalian cells modulates many cellular responses including transcription, chromatin dynamics, differentiation and cell death, in addition to playing a key role in the response to DNA damage [6, 7]. Once activated, the PARP proteins catalyze the formation of ADP-ribose polymers onto acceptor proteins. In addition to this direct covalent modification, some proteins have a high affinity for the polymers themselves and this is exploited in some settings, for instance in DNA repair, for the control of the localization and function of different repair proteins [8]. The radiosensitization effects of PARP inhibitors (PARPi) have been shown to be specific to cells in the S-phase of the cell cycle [9] and are due to the collision of the persisting single strand breaks with replication forks and the formation of a lethal DNA double strand break [10, 11].

This cell cycle specificity of the impact of PARPi could be of particular benefit in the treatment of cancer cells that often show radioresistance during the S phase of the cell cycle [12] and PARPi might result in increased efficiency of radiotherapy in rapidly growing tumors with a high S-phase content. Changes in the tumour microenvironment brought about by PARPi can also have an impact on responses to radiotherapy. For instance, vasoactivity has been reported for some PARPi that may modulate the levels of hypoxia within tumours [13, 14]. Indeed, as hypoxic cells are more radioresistant than oxic cells and intra-tumoural hypoxia can be a significant source of treatment failure following radiotherapy, the reoxygenation of hypoxic tumors could be of therapeutic benefit. Indeed, Liu et al. showed that inhibition of PARP activity can sensitize hypoxic cancer cells and the combination of ionizing radiation-PARP inhibition has the potential to improve the therapeutic benefit of radiotherapy [15]. Therefore, there is a strong rational for the use of PARPi in association with radiation therapy, in particular to counteract the radioresistance of cancer cells in S phase and that of hypoxic cells.

PARPi have shown encouraging results in preclinical trials as a single therapeutic agent in *BRCA2* mutation carriers of breast and ovarian cancers and also in combination trials with chemotherapeutic agents and radiotherapy [16]. Clinical trials of PARPi in combination with radiation therapy are ongoing for instance, phase I and I/II trials of veliparib (ABT-888) and olaparib in combination with radiotherapy are ongoing for brain, lung, head and neck, pancreas and breast cancers [17]. Recently, Quilez-Perez and colleagues [18] have reported that the inhibition of PARP activity using DPQ (3,4-dihydro-5-[4-(1-piperidinyl)butoxyl]-1(2H)-isoquinolinone) was capable of controlling HCC xenograft growth, protecting against diethylnitrosamine-induced hepato-carcinogenesis and also preventing tumour vasculogenesis by the transcriptional regulation of both transcription factors and the expression of genes involved in tumour progression. Munoz-Gamez et al. [19] have shown that the PARPi ANI (4-amino-1,8-naphthalimidecan) enhanced cell growth inhibition induced by doxorubicin in human hepatoma cell lines. Due to the strong rational of PARPi in combination with radiation therapy and these promising effects of PARPi on tumour growth in HCC models (reviewed in [20]), our study was aimed to assess the potential of this combined treatment strategy in a panel of eight liver cancer cell lines and primary hepatocytes.

We first characterized the expression levels of several of the PARP family members at the mRNA level, PARP-1 protein levels and PARP activity. We then assessed differences in repair capacities between cell lines using an *in vitro* DNA repair assay and finally we evaluated the cytotoxic potential of the PARPi ABT-888 as a single agent treatment and in combination with ionizing radiation in hepatoma cells.

## Methods

### Cell culture and drug treatment

HepG2 (ATCC HB-8065), HepG2 2.2.15 (kindly provided by Prof. G. Acs, The Mount Sinai Medical Center, New York, NY, USA), Huh7 (kindly provided by Dr. C. Seeger, Fox Chase Cancer Center, Philadelphia, PA, USA), FOCUS (kindly provided by Dr. J. R. Wands, Rhode Island Hospital, Providence, RI, USA), Hep3B (ATCC HB-8064), PLC-PRF-5 (ATCC CRL-8024) HCC cells and SKHep1 (ATCC HTB-52) adenocarcinoma cells were maintained in DMEM/F-12 medium with 10% fetal bovine serum and 1% penicillin/streptomycin. Geneticin at 100 μg/ml was added to the HepG2 2.2.15 cells’ medium. Primary human hepatocytes (PHHs) were isolated from surgical liver specimens obtained during partial hepatectomy. Informed consent was obtained from each patient, and the procedure was approved by the local Ethics Committee CPP Sud-Est IV (Agreements of the French Ministry of Education and Research n° AC-2013-1871 and n° DC-2013-1870, ISO certification n° NFS 96 900). HepaRG hepatoma cells (established in our laboratory, [21]) and PHHs were maintained in William’s E medium with 10% fetal bovine serum and 1% penicillin/streptomycin supplemented with hydrocortisone sodium succinate. ABT-888 (Veliparib) (Abbott Laboratories, Abbott Park, Illinois, USA) was dissolved in ultrapure water and kept as a 4 mmol/L stock solution in aliquots at -20°C. Doxorubicin (Caelyx) was kept as a stock solution at 2 mg/ml in a pegylated liposomal formulation at 4°C.

### Quantitative real-time polymerase chain reaction (PCR)

Total RNA was isolated from three batches of each liver cell line and PHHs by the RNAble (Eurobio, Courtaboeuf, France) extraction method. An equal amount of RNA was reverse-transcribed to cDNA. Real-time PCR was performed with SYBRgreen technology using the KAPA SYBR FAST qPCR kit (Clinisciences, Nanterre, France) following the manufacturer’s protocol. All values were normalized for the glyceraldehyde 3-phosphate dehydrogenase (*GAPDH*) expression levels. The relative quantification of gene expression was determined using the comparative Ct method. All samples were assayed in triplicate, and mean expression values were used. Primers (Table 1) were designed using the PrimerQuest tool [22] using mRNA NCBI reference sequences.

**Table 1:** **Primer pairs used for gene expression studies and sequencing for single nucleotide polymorphisms genotype studies**

Primer name	Primer sequence (5′-3′)	Tm (°C)	NCBI reference sequence
PARP-1 forward	GCCGAGATCATCAGGAAGTATG	62	[GenBank:NM_001618.3]
PARP-1 reverse	ATTCGCCTTCACGCTCTATC	62	
PARP-2 forward	GTGGAGAAGGATGGTGAGAAAG	62	[GenBank:NM_001042618.1]
PARP-2 reverse	CTCAAGATTCCCACCCAGTTAC	62	
PARP-3 forward	GAGACTACCAGCTTCTCAAGTG	62	[Genbank:NM_001003931.2]
PARP-3 reverse	GTTGCTGCCAGTCTGTTCTA	62	
PARG forward	GTCACCGATGGATGTGGATAA	62	[GenBank:NM_003631.2]
PARG reverse	GGGAGGAACTACCATCTTCTTG	62	
GAPDH forward	CAAGAGCACAAGAGGAAGAGAG	62	[GenBank:NM_001256799.1]
GAPDH reverse	CTACATGGCAACTGTGAGGAG	62	
rs8679 forward	TGTGGGAAGACCAAAGGAAG	52	
rs8679 reverse	ATAGAGAAGGCATCTGCATTTTTAAT	52	
rs1136410 forward	TTTGCCATTCACTGTGTTGG	55	
rs1136410 reverse	TTAATGTCAGTTTTGAGCTTCTCATAG	55	

### Western immunoblot analysis

Western blots of total cell extracts were carried out as previously described [11] using anti-PARP-1 C2-10 primary antibody (BD Biosciences, Benelux, N.V) diluted 1: 1000 in TBS containing 5% milk, Tween-20 (TBS-MT) or anti-pADPr 10H primary antibody (AbCAM, Paris, France) diluted 1 : 500 in TBS-MT.

Immunoblots were developed using the enhanced chemiluminescence detection system (GE Healthcare life sciences, Glattbrugg, Switzerland) following the manufacturer’s protocol and autoradiography. Semi-quantification was made by densitometry analysis using Image J software (NIH).

### Genotyping of PARP-1 single nucleotide polymorphisms rs8679 and rs1136410

The genotypes of the PARP-1 single nucleotide polymorphisms (SNPs) rs8679 and rs1136410 were determined by direct sequencing of 10 ng genomic DNA (see Table 1 for primers and PCR conditions).

### Clonogenic survival assays

Cells were diluted serially to appropriate concentrations and seeded into 25 cm^2^ vented flasks at a cell density of 80 cells/cm^2^ in triplicate per data point and allowed to adhere for 4 h before treatment (37°C, 5% CO_2_). Cells were then exposed or not to ABT-888 (10 μmol/L, 2 h) and subsequently grown for 14 days, fixed in methanol and stained with 0.05% Coomassie brilliant blue in 3:1:6 ethanol/acetic acid/water. Colonies larger than 50 cells were counted by eye and the colony count relative to mock-treated cells (Surviving fraction S) was determined. In radiation survival assays, ABT-888 (10 μmol/L) was introduced 1 h before irradiation. Irradiation was performed using a Caesium-137 source (IBL-637 or a GSR D1) at room temperature at a dose-rate of 0.012 Gy/s [11]. ABT-888 was removed 1 h after irradiation. The colony count relative to mock-treated cells (S) was adjusted for best fit to the classical linear-quadratic equation (Ln S = - α.D - β.D^2^) where D is the radiation dose and α and β adjustable parameters characterizing the radiosensitivity. The mean lethal dose (D_37_), i.e., the dose of radiation leaving 1/e = 0.37 survival, was calculated from the α, β values. Calculations were made through nonlinear least-squares regression taking all data points into account, using Kaleidagraph software (Synergy Software, Reading, Pennsylvania).

### DNA excision/synthesis assay

Cells (3.10^6^ cells) were pelleted by centrifugation and suspended in DMEM/F-12 medium completed with 10% fetal bovine serum and 10% DMSO and frozen at -80°C until the preparation of cell extracts. Cell nuclear extracts were prepared as previously described [23]. Protein content was in the 1–3 mg/mL range. For the plasmid microarray assay, hydrogel slides were purchased from BMT Biosystem (Woodbridge, CT, USA). Six types of DNA lesions were generated in the plasmids before their spotting: photoproducts (cyclobutane pyrimidine dimers and (6–4) photoproducts; CPD-64 plasmid), 8-oxoguanine (8oxoG plasmid), alkylated bases (AlkB plasmid), T-T inter- and intra-strand crosslinks psoralen adducts (Pso plasmid), abasic sites (AP sites; AbaS plasmid) and cytosine and thymine glycols (Glycol plasmid). Each type of modified plasmid was then diluted in non-modified plasmid so we obtained 3 solutions of identical DNA concentration (40 μg/mL) but with 3 different ratios of lesion/DNA (ratios ½, ¾ and 1). On all microarrays each plasmid solution was spotted in duplicate except the control that was deposited 9 times. Excision/synthesis reaction was conducted on the modified plasmid arrays as described in [24] at a final protein concentration of 0.2 mg/mL for all samples. Each extract was tested in duplicate. Images were acquired by scanning the support at 532 nm at a 10 μm resolution using an InnoScan710AL scanner (Innopsys, France). Total spot fluorescence intensity was determined using the Mapix software (Innopsys). Duplicate data were normalized using NormalizeIt software [23]. Each sample was characterised by 6 fluorescence intensity values corresponding to the repair of the 6 DNA lesions represented on the biochip.

### Statistical analysis

For gene and protein expression analyzes we applied a base-10 logarithmic transformation to the values. Data are expressed as means ± standard error of mean (SEM). P values were calculated by independent t-test between two groups using GraphPadPrism4 software (GraphPad, La Jolla, CA, USA) *, p < 0.05; **, p < 0.01; ***, p < 0.001. Correlation analyzes were made by Pearson test. For total fluorescence intensity of excision/synthesis assays, we applied a base-10 logarithmic transformation to the values. Data are expressed as means ± standard deviation (SD). P values were calculated by independent t-test between two groups.

## Results

### mRNA expression of *PARP-1*, *PARP-2*, *PARP-3*and *PARG*in liver cells

**Figure 1:**
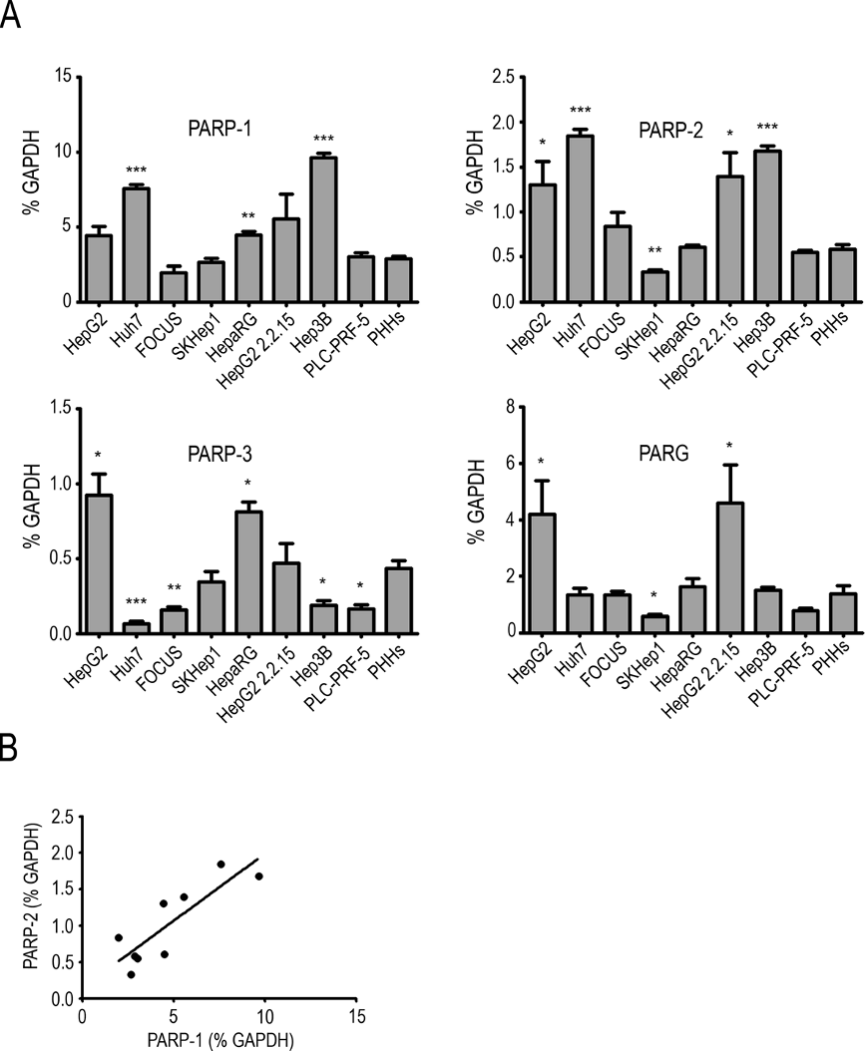
***PARP-1,*** ***PARP-2,*** ***PARP-3,*** ***PARG*** **genes expression in liver cancer cell lines and primary human hepatocytes. (A)** Gene expression profiles were analyzed by real-time quantitative PCR, expressed as % of *GAPDH* expression. **(B)** Pearson correlation analysis showed a significant positive correlation of *PARP-1* vs. *PARP-2* gene expression (Pearson correlation coefficient r = 0.8482 , two-tailed p value = 0.0039).

Before analyzing the cell killing effects of a PARPi and ionizing radiation, we investigated the expression of three members of the PARP family and poly(ADP-ribose) glycohydrolase (PARG) and PARP enzymatic activity profiles in a panel of liver cancer cell lines and PHHs: HepG2, Huh7, FOCUS are hepatoma cell lines, SKHep1 an adenocarcinoma cell line, HepaRG a non-tumorigenic hepatoma cell line and HepG2 2.2.15 is a clone of HepG2 cells harboring in its genome four tandem copies of the hepatitis B virus (HBV) genome. Hep3B is a hepatoma cell line containing a naturally integrated HBV genome and PLC-PRF-5 is a hepatoma cell line expressing HBV surface antigen (HBsAg). Some considerable variation in the mRNA expression levels was seen within the panel of cell lines examined compared to the PHHs (Figure 1A). We found a significant correlation between *PARP-1* and *PARP-2* mRNA expression (Pearson correlation coefficient r = 0.8482, two-tailed p value = 0.0039, Figure 1B); however no correlation was found between the expression of the other genes at the mRNA level.

### PARP-1 protein expression and PARP activity in liver cells

**Figure 2:**
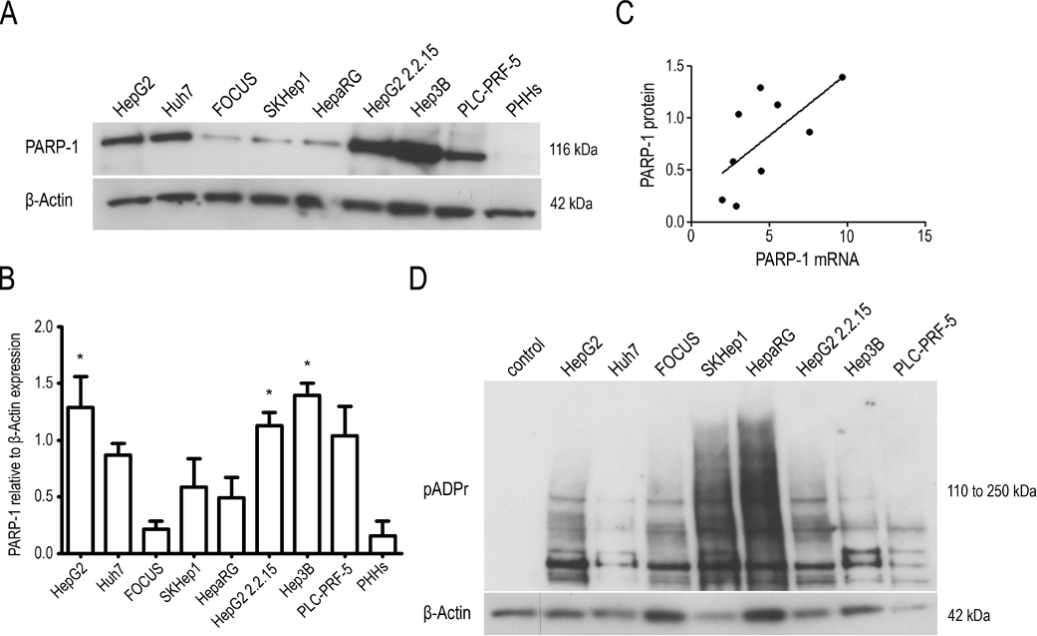
**PARP-1 protein expression and PARP activity in liver cells. (A)** PARP-1 protein expression was analyzed by western blotting. **(B)** Densitometry analyses of PARP-1 expression were performed on three independent western blotting experiments. **(C)** Pearson correlation analysis was performed on the mean of gene and protein expression values and showed a significant positive correlation of PARP-1 protein vs. *PARP-1* mRNA expression (Pearson correlation coefficient r = 0.6840, one-tailed p value = 0.0252). **(D)** PARP activity was measured by western blot analysis of pADPr after cells were treated with 5 μg/ml doxorubicin. HepG2 cells non-treated with doxorubicin were used as a control.

To test whether the mRNA expression correlates with protein levels, we next measured PARP-1 protein levels in the same panel using western blot analysis (Figure 2A). Densitometry analyses were performed on three independent experiments (Figure 2B) and a significant positive correlation was found between PARP-1 protein and mRNA expression (Pearson correlation coefficient r = 0.6840, one-tailed p value = 0.0252, Figure 2C). It was noted that the HepG2 2.2.15 and Hep3B cells, that both carry an integrated HBV genome in their own genome, exhibited the highest expression levels and PLC-PRF-5 cells which express the viral antigen HBsAg also have high PARP-1 protein levels. Almost all liver cancer cell lines have a higher PARP-1 protein expression than that observed in PHHs. Differences in PARP activity between the eight liver cell lines were measured by treating the cells with doxorubicin for 2 h to induce ADP-ribose polymers (pADPr) synthesis and analyzing the pADPr generated by western blot (Figure 2D). As has already been reported in other systems [25], PARP activity did not reflect PARP-1 expression profiles, and we did not find any correlation between PARP activity and either PARP-1 protein or mRNA levels. The *PARP-1* Val762Ala (T2444C) rs1136410 SNP has been associated with a reduced ADP-ribosylation activity and cancer susceptibility [26, 27] and the *PARP-1* 3′ UTR rs8679 T > C SNP is associated with bladder and breast cancer risk [28]. All the cell lines were homozygous for the T allele of both SNPs except HepG2 and HepG2 2.2.15 that were heterozygous for both (Table 2). No consistent differences in *PARP-1* mRNA or protein expression and PARP activity were noted between these two lines and the others.

**Table 2:** **Genotypes of PARP-1 single nucleotide polymorphisms rs1136410 and rs8679 in liver cancer cell lines**

Cell line	rs1136410 genotype	rs8679 genotype
HepG2	T/C	T/C
HepG2 2.2.15	T/C	T/C
Huh7	T/T	T/T
FOCUS	T/T	T/T
SKHep1	T/T	T/T
HepaRG	T/T	T/T
Hep3B	T/T	T/T
PLC-PRF-5	T/T	T/T

### Liver cancer cells have different sensitivity to ABT-888 given as a single agent treatment

**Figure 3:**
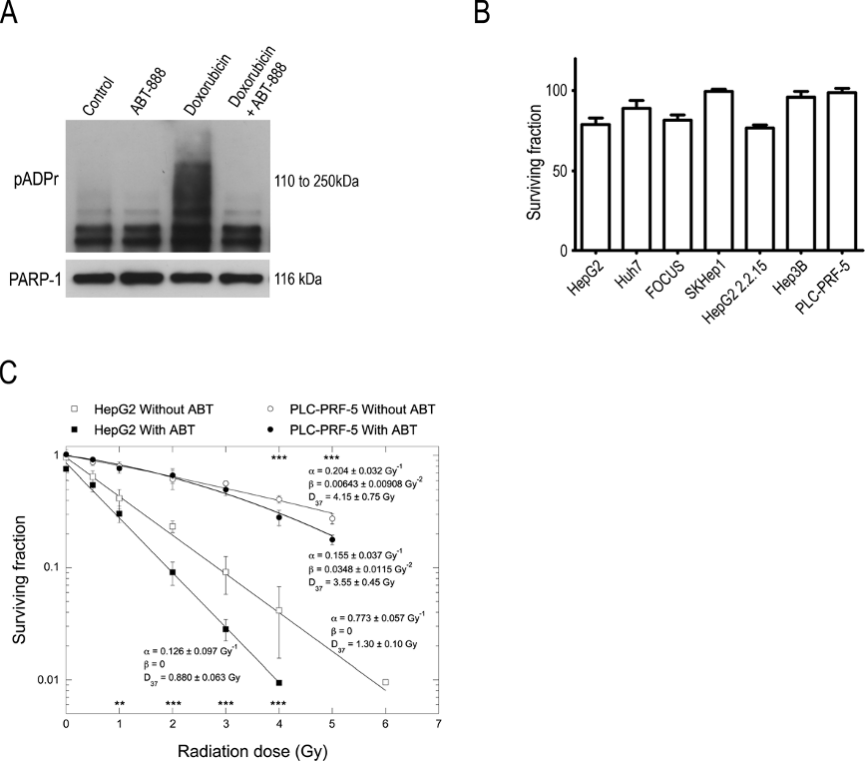
**Effect of the ABT-888 treatment and/or radiation treatment in liver cancer cell lines. (A)** Inhibition of PARP activity was measured by western blot analysis of pADPr levels after HepG2 cells were treated with 5 μg/ml doxorubicin and/or 10 μM ABT-888 for 2 h. Control treatment was the solvent of ABT-888. **(B)** Effect of ABT-888 on clonogenic survival of liver cancer cell lines. Cells were treated with 10 μM ABT-888 for 2 h and allowed to grow for 14 days after treatment. **(C)** Radiation survival of HepG2 (two independent experiments, each in triplicate) and PLC-PRF-5 cells (one experiment in triplicate) without (open symbols) or with (full symbols) ABT-888. The curves were drawn for best fit to the linear-quadratic equation (see Methods). To determine whether the drug-induced modification of radiation response was significant or not, a non-parametric Mann–Whitney U-test was performed on paired sets of data. **, p < 0.001; ***, p < 0.0001 using StatEL (AD Science, Paris, France).

To verify the effective inhibition of PARP activity by ABT-888 in liver cells HepG2 cells were treated for 2 h with doxorubicin to induce pADPr synthesis in the presence or absence of 10 μM ABT-888 and pADPr formation analyzed by western blot. No pADPr was detected in cells co-treated with ABT-888 and doxorubicin, showing that under these conditions PARP activity was inhibited (Figure 3A). We then analyzed the effect of ABT-888 as a single agent treatment on clonogenic survival of the seven liver cancer cell lines in our test panel (Figure 3B). Of these cells, FOCUS, HepG2 2.2.15 and HepG2 were sensitive to the cell killing effects of ABT-888 alone whilst Hep3B and Huh7 cells showed a lower sensitivity. Based on the observations and the PARP activity measured we selected the HepG2 (intermediate PARP activity and sensitive to ABT-888) and PLC-PRF-5 (low PARP activity and resistant to ABT-888) cell lines for the further experiments combining radiation exposure with ABT-888 treatment.

### Liver cancer cells have different excision/synthesis DNA repair capacity

**Figure 4:**
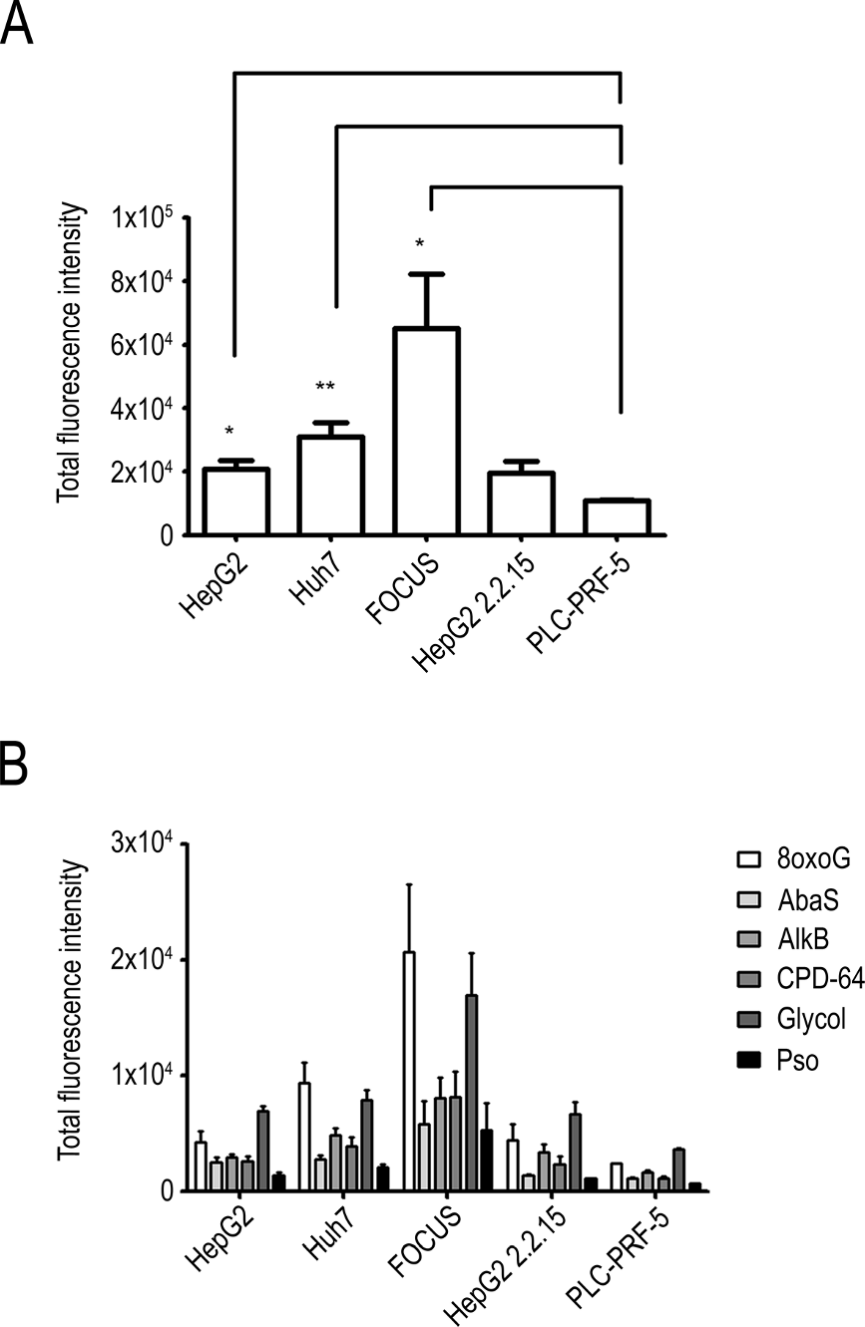
**Excision/synthesis abilities of liver cancer cells. (A)** Total excision/synthesis abilities were analyzed using damaged plasmids microarray containing six DNA damage types and by adding total fluorescence intensity of all DNA lesions. **(B)** Excision/synthesis abilities for each type of DNA lesion (cyclobutane pyrimidine dimers and 6–4 photoproducts (CPD-64), T-T inter- and intra-strand crosslinks psoralen adducts (Pso), 8-oxoguanine (8oxoG), alkylated bases (AlkB), abasic sites (AbaS) and cytosine and thymine glycols (glycol).

The PARP proteins are involved in several DNA repair pathways and thus PARP inhibition could impact on the repair of different DNA lesions. In order to evaluate the constitutive inter-line variation in repair capacity we used a novel excision/synthesis repair assay that makes use of a microarray on which plasmids are spotted that contain different types of DNA damage: CPD and 6-4PPs, Pso lesions that are typically repaired by nucleotide excision repair; 8oxoG, AlkB, AbaS and glycol lesions that are typically repaired by the base excision repair pathway. We first analyzed the total excision/synthesis capacities of each cell line by assessing the total fluorescence values for all the “damaged” plasmids. Using this endpoint, we observed that FOCUS had the highest levels of overall repair capacity, with HepG2, Huh7 and HepG2 2.2.15 cells having intermediate capacities. Conversely, PLC-PRF-5 had a significantly lower capacity compared to the other cells examined (Figure 4A). All the cell lines presented a similar profile of excision/synthesis repair for the different DNA lesion types assessed with a high capacity to repair 8oxoG and glycol modified bases, a lower capacity for AlkB, CPD and 6-4PPs and lower capacities for the repair of AbaS and the lowest seen for Pso lesions (Figure 4B). The low capacity of PLC-PRF-5 cells to repair DNA damage as assessed by this excision/synthesis assay is intriguing in the light of the apparent resistance to the cell killing effects observed after the treatment of these cells with ABT-888 as a single agent described above.

### ABT-888 sensitizes liver cancer cells to the cell killing effects of ionizing radiation

In order to assess the potential of ABT-888 as a radiosensitizer in liver cancer cells we treated HepG2 and PLC-PRF-5 cells with ABT-888 for 2 h and exposed cells to ionizing radiation 1 h after the addition of the PARPi and analyzed cell survival in a clonogenic assay. ABT-888 sensitized both cell lines to the cell killing effects of ionizing radiation . Based on the ratio of the D_37_ values ABT-888 enhanced the radiation susceptibility of HepG2 and PLC-PRF-5 cells by 1.48 ± 0.22 and 1.17 ± 0.36, respectively (Figure 3C).

## Discussion

Hepatocellular carcinoma is one of the most severe cancers worldwide and curative treatments can be offered to a limited number of patients. In this study, we investigated the potential of PARPi to sensitize liver cancer cells to ionizing radiation. Knowing that *PARP-1* mRNA levels are up-regulated in several cancer types [29], we compared the mRNA expression profiles of *PARP-1*, *PARP-2* , *PARP-3* and *PARG* in eight liver cancer cell lines to that of PHHs. *PARP-1* and *PARP-2* mRNA expression appear to be up-regulated in most of the liver cancer cell lines studied in comparison with PHHs although not all the differences in expression were significant. The expression of *PARP-1* mRNA correlated well with the *PARP-2* mRNA expression. This similar expression profile could reflect shared functions in the base excision pathway [6]. In contrast, *PARP-3* mRNA was down-regulated in cancer cells compared to normal cells. Only two cell lines showed significant *PARP-3* up-regulation. Interestingly, Rouleau and colleagues [30] showed in the Cynomolgus monkey that PARP-3 protein is not expressed in hepatocytes and its expression is restricted to bile ducts cells. *PARG* mRNA levels are only significantly higher in two cancer cell lines. These expression profiles are consistent with what it was already observed in breast tumors [31]. Recently, Ko and colleagues [32] have shown that PARPi can enhance HBV replication and we found high levels of *PARP-1* mRNA and protein and *PARP-2* mRNA in cell lines harboring integrated HBV genome (although HepG2 2.2.15 cells originated from HepG2 cells and both express similar levels of the *PARP-1* gene and protein). These observations would suggest that particular attention needs to be given to patients who are infected with HBV and are on clinical trials involving PARPi, and it needs to be established whether PARPi can enhance HBV replication in such patients. Several studies have examined whether correlations exist between *PARP-1* mRNA levels, protein levels and PARP enzymatic activity. In the panel of liver cells examined we found a correlation between mRNA and protein expression which is generally not a common feature for instance in breast tumors [31] but not between protein expression and PARP activity as has already been observed [25]. No correlation was found between the rs1136410 and rs8679 genotypes and PARP-1 expression and PARP activity although only 2 lines carried the variant alleles of these two SNPs which limited the possibility to detect any such genotype specific differences in activity or expression.

The presence of PARP-1 at the protein level in liver cancer cells and detected poly(ADP-ribosyl)ation activity allowed us to investigate the effects of PARPi in these cell lines. Interestingly, the different liver cancer cell lines analyzed showed differential sensitivity to the PARPi ABT-888 used as a single agent treatment. Zhang et al. [33] have also observed liver cell lines with differential sensitivity to the PARPi olaparib. In a first attempt to investigate the underlying reasons for this differential sensitivity we assessed the overall capacity of the cells to carry out the excision/synthesis step for a number of different DNA lesions that are repaired via the base excision repair and nucleotide excision repair pathways. Using this assay we found that the cell lines had some considerable variation in repair capacity for the different lesions with the PLC-PRF-5 cells having very low capacity. This result would suggest that the apparent resistance to the cell killing effects of ABT-888 seen in the clonogenic survival assays may originate from the over-expression of repair activities such as DNA double strand break repair that cannot be assessed using this *in vitro* assay.

Based on these profiles, we selected one cell line sensitive to the cell killing effects of ABT-888 used as a single agent treatment and having high levels of PARP activity and one cell line resistant to ABT-888 under these conditions with low levels of PARP activity to compare their response to the combination of ABT-888 and ionizing radiation. Based on the D_37_ values the radiation susceptibility of HepG2 cells was 2.3-fold higher than that of PLC-PRF-5 cells in the absence of ABT-888. The PARPi produced a radiosensitizing effect that was significantly higher in HepG2 (1.48 ± 0.22) than in PLC-PRF-5 cells (1.17 ± 0.36). These different responses could reflect the differential sensitivity of the two cell lines to ABT-888. PARPi were initially shown to be cytotoxic for cells lacking a component of homologous recombination pathway (reviewed in [8]). Further investigations into the genetic background of sensitive and resistant cells will be needed to provide a better understanding of the different responses to the combination of PARPi with ionizing radiation. Our results suggest that the response to this combination treatment might depend on cells’ capacities to repair DNA damage and assessing DNA repair capacities of tumor cells from patients undergoing clinical trials of PARPi could help to adjust radiation therapy schedules.

## Conclusions

Altogether, our results confirmed the strong potential of PARPi to enhance ionizing radiation in liver cancer cells letting us consider preclinical mice studies and clinical trials of such combination treatment.

## Abbreviations

PARP: Poly(ADP-ribose) polymerase
HCC: Hepatocellular carcinoma
PARPi: PARP inhibitors
DPQ: 3,4-dihydro-5-[4-(1-piperidinyl)butoxyl]-1(2H)-isoquinolinone
ANI: 4-amino-1,8-naphthalimidecan
PHHs: Primary human hepatocytes
PCR: Polymerase chain reaction
*GAPDH*: Glyceraldehyde 3-phosphate dehydrogenase
SNP: Single nucleotide polymorphism
CPD-64: Cyclobutane pyrimidine dimers and (6-4) photoproducts
8oxoG: 8-oxoguanine
AlkB: Alkylated bases
Pso: Psoralen adducts
AbaS: Abasic sites
PARG: Poly(ADP-ribose) glycohydrolase
HBV: Hepatitis B virus
HBsAg: HBV surface antigen
pADPr: ADP-ribose polymers.
